# Supplementary material for: WDR11‐mediated Hedgehog signalling defects underlie a new ciliopathy related to Kallmann syndrome
Source: EMBO Rep. 2017 Dec 20;19(2):269–89. doi: 10.15252/embr.201744632 (PMC5797970; doi:10.15252/embr.201744632)
Supplement: Supplementary file 1 — Appendix [file EMBR-19-269-s001.pdf]

Appendix

WDR11-mediated Hedgehog signalling defects underlie a new ciliopathy related to Kallmann syndrome. Kim et al.

Table of Content

p. 2	Appendix Text	Detailed description of the clinical case.
p. 4-5	Appendix Table S1	Primers used to study Wdr11 knockout mouse and the human and mouse GnRH cell lines.
p. 6-7	Appendix Table S2	Primers and human mutations of WDR11 used in the study.
p. 8-9	Appendix Table S3	Growth parameters of the mice used to calculate BMI.
p.10	Appendix Table S4	Growth parameters of the patients with WDR11 mutation MT.
p.11	Appendix Figure S1	Wdr11 knockout strategy and expression profiles.
p.12	Appendix Figure S2	Wdr11 co-localises with GnRH neurones.
p.13	Appendix Figure S3	Wdr11 KO disrupts the embryonic migration of GnRH neurons and pituitary hormone production.
p.14	Appendix Figure S4	Wdr11 mutant mice show delayed growth and development, reproductive dysfunction and obesity.
p.15	Appendix Figure S5	Knockdown of wdr11 in zebrafish.
p.16	Appendix Figure S6	The effects of Hh signalling in the intracellular localisation of WDR11 and GnRH neuronal cell motility.
p.17	Appendix Figure S7	Pedigree and sequence analyses of WDR11 mutation and the defective intracellular localisation of WDR11 mutants.
p.18	Appendix Figure S8	Hh agonist induces GnRH protein expression in vitro.
p.19	Appendix Figure S9	Hh agonist partially rescues ciliogenesis defects in GnRH neuronal cells in vitro.
p.20-24	Appendix Figure Legends	Legends for the Appendix Figures.
p.24	Appendix Movie Legend	Legends for the movie file.

Appendix Text. Detailed clinical description of the patients and the family members.

All subjects described below have normal intelligence.

The index patient (arrow in the pedigree shown in Appendix Fig. S7A)

He was born with normal length (50 cm) and weight (3.43 kg). His developmental milestones were normal, except for slight delay in the development of speech. His mid-parental target height is -2.6 SDS (father's height, 163 cm, mother's height 144 cm). He displayed short stature in childhood and started to gain weight at the age of five (Appendix Table S3, and Fig. 7C in the main text). He was diagnosed with attention-deficit hyperactivity syndrome which was treated with methylphenidate from the age of 8.8 yrs, and anxiety disorder (treated with quetiapine from 11.1 to 11.4 yrs, aripiprazole from 11.9-13.1 yrs and periciazine from 13.1 yrs onwards). During these medications, he gained weight even more rapidly. In two GH stimulation tests (performed at 9.6 years and 9.9 years of age), his peak GH levels following intravenous arginine administration were very low (0.4 μg/l and <0.2 μg/l). In addition, he had low IGF-1 (<3 nM) and IGFBP-3 (1.2 mg/l and 1.3 mg/l) levels. His brain MRI scan findings are described below in detail. He has received growth hormone treatment from the age of 10.3 years, and he responded to the treatment by accelerating growth (Appendix Table S3), and by normalizing his serum IGF-1 and IGFBP-3 levels. He has not developed additional pituitary hormone deficiencies, and his prolactin level is normal. At the age of 14.3 years, his LH peak to GnRH stimulation was prepubertal (4.2 IU/L) and he received a short course of low-dose testosterone therapy to expedite the onset of puberty. Thereafter, his puberty has progressed spontaneously.

The affected brother

He was born with normal length (49 cm) and weight (2.81 kg). His developmental milestones were normal. He started to gain weight after the age of four years (Supplementary Table 3, and Fig. 7C in the main text). At the age of 11.3 years he exhibited very low GH response to arginine stimulation (peak GH, 0.47 μg/l). His brain MRI results are described below in detail. He did not receive GH treatment, however, as his height-for-age, growth velocity and IGF-1 levels were

repeatedly normal. Indeed, at 13.3 years of age, his response to GHRH was normal (baseline <0.05 to stimulated value of 11.8 μg/l) suggesting the presence of pituitary reserves for GH production. He has not developed clinical or biochemical evidence for additional pituitary hormone deficiencies. He used orlistat from the age of 13.3 years, and metformin from the age of 13.5 years. At 13.7 yrs, being severely overweight, he displayed impaired glucose tolerance in oral glucose tolerance test. His puberty has started normally.

Other family members and the maternal uncle

The mother is short (144 cm), has normal weight (50 kg), and normal BMI (24.1 kg/m^2). She has L-T4 medication, which has been started in the primary healthcare. Her brain MRI is normal. The length of the father is 163 cm; he has normal weight (66.5 kg), corresponding to BMI of 25 kg/m^2; his MRI of the pituitary gland and hypothalamic region was normal. The sister of the boys does not have signs or symptoms of pituitary hormone deficiencies, her adult height is 150 cm (-3 SDS), and her weight is +60% as an adult (BMI 29.3 kg/m^2). The MRI scan of her pituitary gland and cellar region was normal. The maternal uncle of the index patient is short (155 cm), has normal weight (66.5 kg) and BMI consistent with overweight (27.7 kg/m^2). The MRI scan of the pituitary gland and cellar region was normal. He has five children.

Imaging analysis

The MR images were evaluated by neuroradiologist (NB) for the appearance of the olfactory tracts and the hypophyseal area or other brain abnormalities. The pituitary stalk was present in the index case (older of the brothers), but truncated in the affected, younger brother. The neurohypophysis (posterior pituitary bright spot) was present in abnormal position and the adenohypophysis was hypoplastic in the both patients. No other midline abnormalities were present. Other cranial features, as assessed by MR imaging, were also normal.

Appendix Table S1. Primers used to study *Wdr11* knockout mouse and the human and mouse GnRH cell lines

A. Primers used for confirmation of gene trap insertion and genotyping

Primer	Sequence	Length (bp)	Strategy
KBW205-3	5'-GGGATTTCACGGAACCCAACTGCTG-3'	25	Long PCR to identify the 5' integration site
KBW205-4	5'-CTCATCCTAGGGGCATTGTGGATTC-3'	25	
LPBL	5'-GGATCCGGAACCCTTAATATAACTTCG-3'	27	
KBW205-1	5'-CCTACACCGTAAACTTCAAGG-3'	21	RT-PCR after 5'-RACE
KBW205-2	5'-TCTCAACGCGCACAACAAGG-3'	20	
Z-1	5'-GGGTTACCCAACTTAATCG-3'	19	Genotyping of trap allele (LacZ gene)
Z-2	5'-TGTGAGCGAGTAACAACCCG-3'	20	
KBW205-5	5'-ATGGCCTGGGATTTGATGACC-3'	21	Genotyping of trap and WT allele from 5' integration site
KBW205-6	5'-AGGTTGACCCAATCTCTGCTC-3'	21	
SA-5AS	5'-GGGCAAGAACATAAAGTGACC-3'	21	
KBW205-7	5'-AGAGTGGTCTGAGAGGAAAGG-3'	21	
KBW205-8	5'-GAAAGAGAAACACGGACAGAG-3'	21	
Intron2F	5'-CCCTGTCTCGAAAAACCAAA-3'	20	
KBW205-9	5'-TTGGCCATGCCTAGGAAAGTC-3'	21	PCR to identify the 3' integration site
KBW205-10	5'-TAATTAGGCGGGTGGATAGCG-3'	21	
KBW205-11	5'-TGGGGTTACAAGTCAGCATGC-3'	21	
pSP72-2	5'-ATAGTTAAGCCAGCCCCGAC-3'	20	
pSP72-3	5'-ATAAGGGCGACACGGAAATG-3'	20	
pSP72-5	5'-TCACGTTAAGGGATTTTGGTC-3'	21	
ori-1	5'-AGAGGCGGTTTGCGTATTGG-3'	20	
ori-2	5'-CAGTGGCGATAAGTCGTGTC-3'	20	
Amp-S	5'-TACAGGCATCGTGGTGTCAC-3'	20	
Amp-AS	5'-AAATGTGCGCGGAACCCCTA-3'	20	
pSP72-8	5'-GTGAAAACCTCTGACACATGC-3'	21	
pSP72-1	5'-GGGCGTGCTTTACTATGCG-3'	19	
pSP72-6	5'-TGTAGGTATCTCAGTTCGGT-3'	20	
SP6	5'-CATACGATTTAGGTGACACTATAG-3'	24	

B. qRT-PCR primers for human genes

Gene	Genebank acc no.	Tm (°C)	Primer sequence	Product size (bp)
GLI1	NM_005269.2	60	F: 5' GCCGTATGTATGTAAGCTCC'3<br>R: 5' ACTGTAGAAATGGATGGTGC'3	156
GLI2	NM_005270.4	56	F: 5' CTCCACGACTACCTCAACCC'3<br>R: 5' GAGAGTGGGGAGATGGACAG'3	101
GLI3	NM_000168.5	59	F: 5'CTTTGCAAGCCAGGAGAAAC'3<br>R: 5'TGTTGGACTGTGTGCCATTT'3	162
EMX1	NM_004097.2	60	F: 5' CTTCGTGAGTGGCTTCCCT'3<br>R: 5' GTGGTTCATGGCCTCGGG'3	94
PTCH1	NM_000264.3	55	F: 5'TGTTCCAGTTAATGACTCCC'3<br>R: 5'ACACTCTGATGAACCACCTC'3	145
WDR11	NM_018117.11	59	F: 5'GGCTCTCCTGGTTCTCCTCT'3<br>R: 5'GCTCCATACTTGAGGCAAGC'3	117
GAPDH	NM_002046.5	59	F: 5'CGAGATCCCTCCAAAATCAA'3<br>R: 5'TTCACACCCATCACAAACAT'3	170
GNRH1	NM_000825.3	59	F: 5'CAGAAACCCAACGCTTCGAA'3<br>R: 5'TCCTTCTGGCCCAATGGATT'3	132

C. qRT-PCR primers for mouse genes

Gene	Genebank acc no.	Tm (°C)	Primer sequence	Product size (bp)
Gli3	NM_000168.5	59	F: 5'CTGCAGTGAGAGTGGACAGG'3<br>R: 5'GTATCCAGTTGTGGGCTGCT'3	239
Emx1	NM_010131.2	59	F: 5'AATCACTACGTGGTGGGAGC'3<br>R: 5'CCCTTCCTCTTCCAGCTTCT'3	129
Emx2	NM_010132.2	59	F: 5'CAGAGAAATGAGGGAGCAGG'3<br>R: 5'TTTGGGTCTTTTATCGTGGG'3	107
Wdr11	NM_172255.3	59	F: 5'CATTTGACCAACCACAGCAC'3<br>R: 5'GACCACGGACGCTAAACATT'3	133
Gapdh	NM_001289726.1	59	F: 5'CGTCCCGTAGACAAAATGGT'3<br>R: 5'GAGGTCAATGAAGGGGTCG'3	129
GnRH1	NM_008145.2	59	F: 5'TCAACCTACCAACGGAAGCT'3<br>R: 5'CCAAACACACAGTCAGCAGT'3	107
Wdr11 exon3/4	NM_172255.3	55	F: 5'TGTGAGATCCAAGAGCACGT'3<br>R: 5'GCACGATGTAATTAGGCGGG'3	109
Fsh	NM_008045.3	59	F: 5'GCCGTTTCTGCATAAGC'3<br>R: 5'CAATCTTACGGTCTCGTATACC'3	135
Lh	NM_008497.2	59	F: 5'CTGTCAACGCAACTCTGG'3<br>R: 5'TAGGTGCACACTGGCTGA'3	145
Gh	NM_008117.3	55	F: 5'ACTGCTTGGCAATGGCTACA'3<br>R: 5'GAGTTCGAGCGTGCCTACAT'3	196
Prl	NM_011164.2	55	F: 5'CTGCCAATCTGTTCCGCTG'3<br>R: 5'CAAGCCCTGAAAGTCCCTC'3	223
Fgfr2	NM_000141.4	60	F: 5'CCCATCCTCCAAGCCGGACTGCCG'3<br>R: 5'GTCTGGGGAAGCTGTAATCTCCTT'3	357

D. q-PCR primers for chromatin immunoprecipitation assays

Gene	Genebank acc no.	Tm (°C)	Primer sequence	Product size (bp)
PTCH1 (GliBS-A)	NM_000264.3	60	F: 5'AGCGCCTGTTTACCCAGGAG3'<br>R: 5'GCTCCTCCGTCTTCTCCCAG'3	396
PTCH1 (GliBS-B)	NM_000264.3	60	F: 5'TATTGCATGCGAGAAGGTTG'3<br>R: 5'GAGAGCGAGCGAAAGAGAAA'3	236

Appendix Table S2. The study of human mutations of WDR11

A. Primers used to confirm the variants by Sanger sequencing

GenBank acc no.	Primers	Primer sequence
NM_018117.11	*WDR11* ex12 forward	5'-ATATGACTCTCTCCCTGGCC-3'
NM_018117.11	*WDR11* ex12 reverse	5'-CCATGTAAACAATGATGAGGCCT-3'
NM_001029864.1	*KIAA1755* ex4 forward	5'-GGCACATGGGAGAGATCAAT-3'
NM_001029864.1	*KIAA1755* ex4 reverse	5'-CTCCACCCAACAGCAGCT-3'
NM_022140.3	*EPB41L4A* ex4 forward	5'-ACAGTTCTGAACCTTGCTGTT-3'
NM_022140.3	*EPB41L4A* ex4 reverse	5'-ACTGATCAACTTCATGCAATGC-3'
NM_001039753.2	*EML6* ex29 forward	5'-ATACCGTTCCTGGGACACAC-3'
NM_001039753.2	*EML6* ex29 reverse	5'-ACTGAACCTGAGCGTTACCA-3'
NM_001037131.2	*AGAP1* ex12 forward	5'-CACTATGTGCCAGCGTGT-3'
NM_001037131.2	*AGAP1* ex12 reverse	5'-TGTGAGTCTCTGATGCAGCC-3'
NM_001282620.1	*GNAI2* ex1 forward	5'-CGTGAGCCTCTGAGAGCAAA-3'
NM_001282620.1	*GNAI2* ex1 reverse	5'-AACTAGCCCTCTGTGGCAC-3'
NM_014228.3	*SLC6A7* ex2 forward	5'-GCCCTGTTCAATTCCAGAGT-3'
NM_014228.3	*SLC6A7* ex2 reverse	5'-CAATGAGTTCTGCCCAAGGT-3'
NM_001037763.2	*COL28A1* ex32 forward	5'-AGGTCTCAGGTTCATCATTGAC-3'
NM_001037763.2	*COL28A1* ex32 reverse	5'-GGATTGTACCCTCCAAACTGA-3'
NM_005181.3	*CA3* ex6 forward	5'-GCTTCCCTGCTTTGATCTTTATT-3'
NM_005181.3	*CA3* ex6 reverse	5'-TGGCCATAGAGCTGTTCAGT-3'
NM_153809.2	*TAF1L* variant forward	5'-ACAAGTATCAGAGTCGGGAGA-3'
NM_153809.2	*TAF1L* variant reverse	5'-CACTCCCAGCATCTTCCTCA-3'
NM_004308.3	*ARHGAP1* ex12-13 forward	5'-CTCCCCTCTAGCCTGCATC-3'
NM_004308.3	*ARHGAP1* ex12-13 reverse	5'-CTTCATGGCCCCTGATGC-3'
NM_001287241.1	*ITM2C* ex3 forward	5'-CGTGTATGACCAGCCTCTCT-3'
NM_001287241.1	*ITM2C* ex3 reverse	5'-CCTAACCGTAGACACCTGCT-3'
NM_001305.4	*CLDN4* variant forward	5'-CGCCCTCGTCATCATCAGC-3'
NM_001305.4	*CLDN4* variant reverse	5'-AGCAGAATACTTGGCGGAGT-3'
NM_004098.3	*EMX2* ex1 variant forward	5'-GCTGCTTCACCATCGAGTC-3'
NM_004098.3	*EMX2* ex1 variant reverse	5'-CACGTACCTTGGAAGCGATG-3'
NM_014247.2	*RAPGEF2* ex23 forward	5'-TCTGCTGCTACACTGTGGAT-3'
NM_014247.2	*RAPGEF2* ex23 reverse	5'-TTAAGAGAACAGAAATGCCTTGG-3'
NM_020816.3	*KIF17* ex15 forward	5'-GCTCTCCATCCTACCCACTT-3'
NM_020816.3	*KIF17* ex15 reverse	5'-CAGACAGAGGCACAGTTCCT-3'
NM_014956.4	*CEP164* ex30 forward	5'-TTGTCTGGAGAAGCAGGGAG-3'
NM_014956.4	*CEP164* ex30 reverse	5'-CAGAGTGGGGCTTACATGGA-3'
NM_005544.2	*IRS1* variant forward	5'-CAGAGTGCCAAAGTGATCCG-3'
NM_005544.2	*IRS1* variant reverse	5'-AAAGAACAGGAAGGGGCAGA-3'
NM_178857.5	*RP1L1* variant forward	5'-TGGGTCCCATGCCATGAC-3'
NM_178857.5	*RP1L1* variant reverse	5'-CCTCCTTCTGGCCCTTCTTT-3'

B. Primers used to generate the *WDR11* mutation c.1610C>T (patient MT)

Primers	Primer sequence
Mutagenesis forward	5’-CTTTCTTTTGCTACCTCAACACTAAACAATATGGGATTAGTGAGA-3’
Mutagenesis reverse	5’-TCTCACTAATCCCATATTGTTTAGTGTTGAGGTAGCAAAAGAAAg-3’
Sequencing forward	5’-TCGTATGTGTCCACCGTTGA-3’

C. Human mutations of *WDR11* analysed in this study

	RSID	Protein	Transcript	Allele Frequency	Location in the protein
M1	rs201051480	p.Arg395Trp	c.1183C>T	.	Linker between WD5 and 6
M2	rs318240760	p.Ala435Thr	c.1303G>A	0.0001322	WD6
M3	rs144440500	p.Arg448Gln	c.1343G>A	0.0001648	WD6
MT	rs761599645	p.Pro537Leu	c.1610C>T	0.00001647	Linker between WD6 and 7
M4	rs318240761	p.His690Gln	c.2070T>A	0.00006607	WD9
M5	rs139007744	p.Phe1150Leu	c.3450T>G	0.0005683	Distal to WD12

Appendix Table S3. Growth parameters of the mice.

A. Mouse BMI at 1 week

Genotype	Age (weeks)	Body length (m)	Body weight (kg)	**BMI (kg/m²)**	**Mean ± SEM**
+/+	1	0.0493	0.00696	2.86	
+/+	1	0.0534	0.00680	2.39	
+/+	1	0.0567	0.00670	2.08	2.66±0.14
+/+	1	0.0486	0.00650	2.75	
+/+	1	0.0470	0.00650	2.94	
+/+	1	0.0485	0.00683	2.90	
+/-	1	0.0460	0.00650	3.07	
+/-	1	0.0450	0.00600	2.90	
+/-	1	0.0430	0.00650	3.52	3.55±0.19
+/-	1	0.0410	0.00660	3.93	
+/-	1	0.0390	0.00600	3.94	
+/-	1	0.0400	0.00630	3.94	
-/-	1	0.0270	0.00560	7.82	
-/-	1	0.0260	0.00520	7.69	
-/-	1	0.0330	0.00590	5.42	6.74±0.55
-/-	1	0.0300	0.00650	5.31	
-/-	1	0.0250	0.00520	8.32	
-/-	1	0.0320	0.00600	5.86	

B. Mouse BMI at 25 weeks

Genotype	Age (weeks)	Body length (m)	Body weight (kg)	**BMI (kg/m²)**	**Mean ± SEM**
+/+	25	0.200	0.0360	0.90	
+/+	25	0.210	0.0380	0.86	
+/+	25	0.190	0.0347	0.96	
+/+	25	0.195	0.0341	0.90	
+/+	25	0.200	0.0370	0.93	0.92±0.02
+/+	25	0.200	0.0380	0.95	
+/+	25	0.190	0.0360	1.00	
+/+	25	0.210	0.0391	0.89	
+/-	25	0.194	0.0400	1.06	
+/-	25	0.180	0.0449	1.39	
+/-	25	0.170	0.0466	1.61	
+/-	25	0.180	0.0486	1.50	1.38±0.07
+/-	25	0.180	0.0434	1.34	
+/-	25	0.170	0.0457	1.58	
+/-	25	0.190	0.0430	1.19	
+/-	25	0.170	0.0398	1.38	
-/-	25	0.150	0.0386	1.72	
-/-	25	0.150	0.0370	1.64	
-/-	25	0.152	0.0400	1.73	1.68±0.02
-/-	25	0.150	0.0369	1.64	
-/-	25	0.150	0.0387	1.72	
-/-	25	0.150	0.0367	1.63	

Appendix Table S4. Growth parameters of the patients with *WDR11* mutation MT.

A. Index patient

Age (yr)	Length (cm)	Height SDS	Weight (kg)	Weight-for-length (DW%)¤
0	50	-0.6	3.43	1%
1.2	73.9	-2.6	9.53	1%
2.1	80.6	-3.3	11.73	7%
5	98	-3.3	16.4	7%
6	102.2	-3.7	19.0	15%
7.3	109.4	-3.5	24.3	30%
8.5	114.7	-3.5	28.5	39%
9.5	120.6	-3.2	33.0	45%
10.6	128.0	-2.7	39.1	49%
11.8	142.2	-1.3	56.0	60%
12.9	149.4	-1.2	84.8	110%
13.9	158	-1.0	106.6	124%
14.8	163.8	-1.0	97.0	84%
16.0	167.1	-1.2	113.0	103%

B. Affected younger brother of the index patient

Age (yr)	Length (cm)	Height SDS	Weight (kg)	Weight-for-length (DW%)¤
0	49	-1.1	2.81	-12%
1.0	71	-2.6	8.63	-2%
1.9	78.5	-3.3	11.15	6%
4	94	-2.7	15.5	9%
5.1	101.5	-2.6	21.2	30%
7.5	117.4	-2.1	36	67%
9	123.3	-2.4	37.2	55%
10	127.5	-2.4	46.2	78%
11.1	135.0	-1.9	68.7	128%
11.9	139.9	-1.7	81.5	145%
12.8[1]	146.7	-1.4	93.5	144%
13.5[2]	151.0	-1.5	107.3	158%
13.8	154.4	-1.3	110.9	149%

¤Weight-for-length is the percentage deviation of weight from the median weight for length and sex (DW%)[74]

Appendix Figure S1.

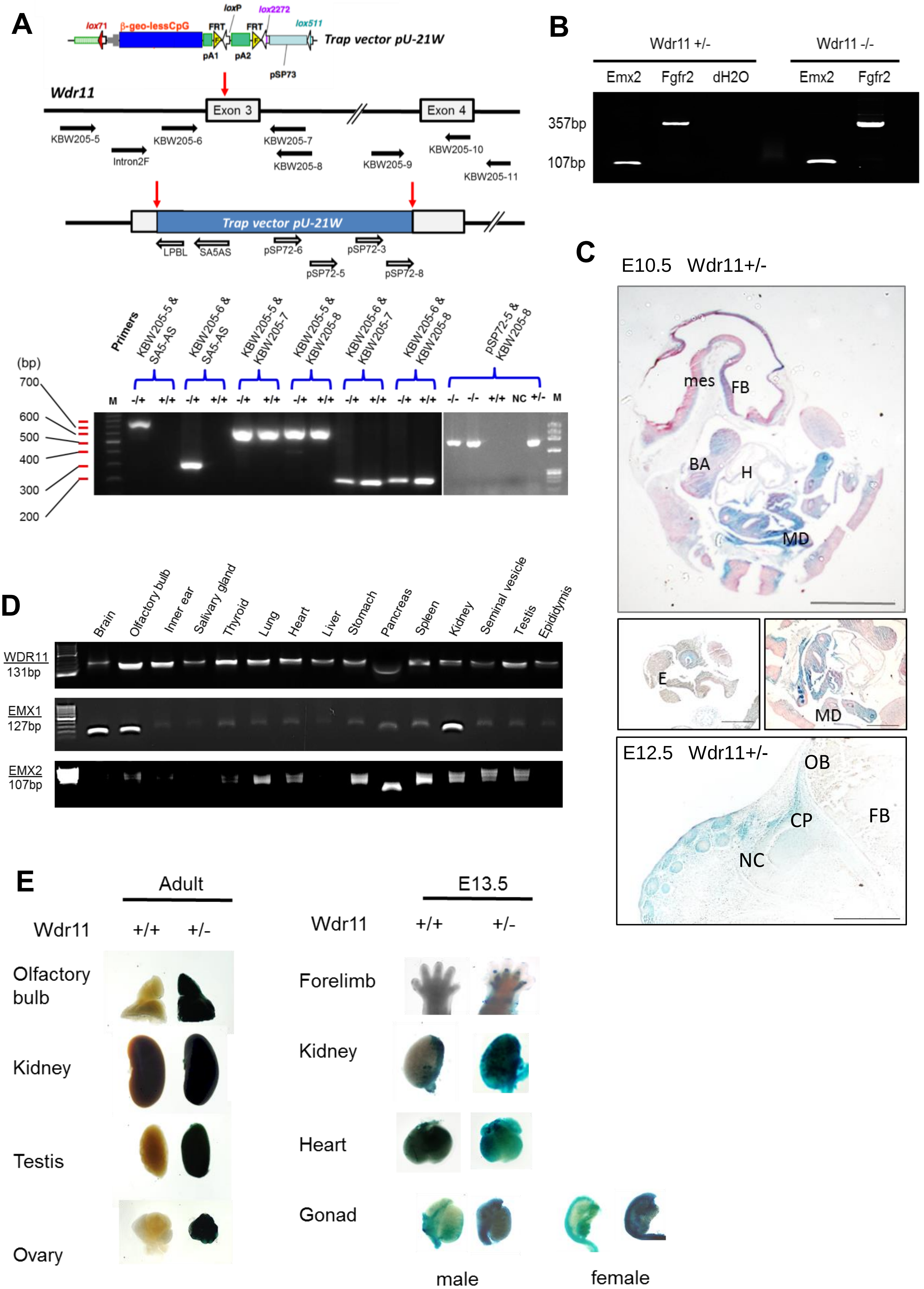

Appendix Figure S2.

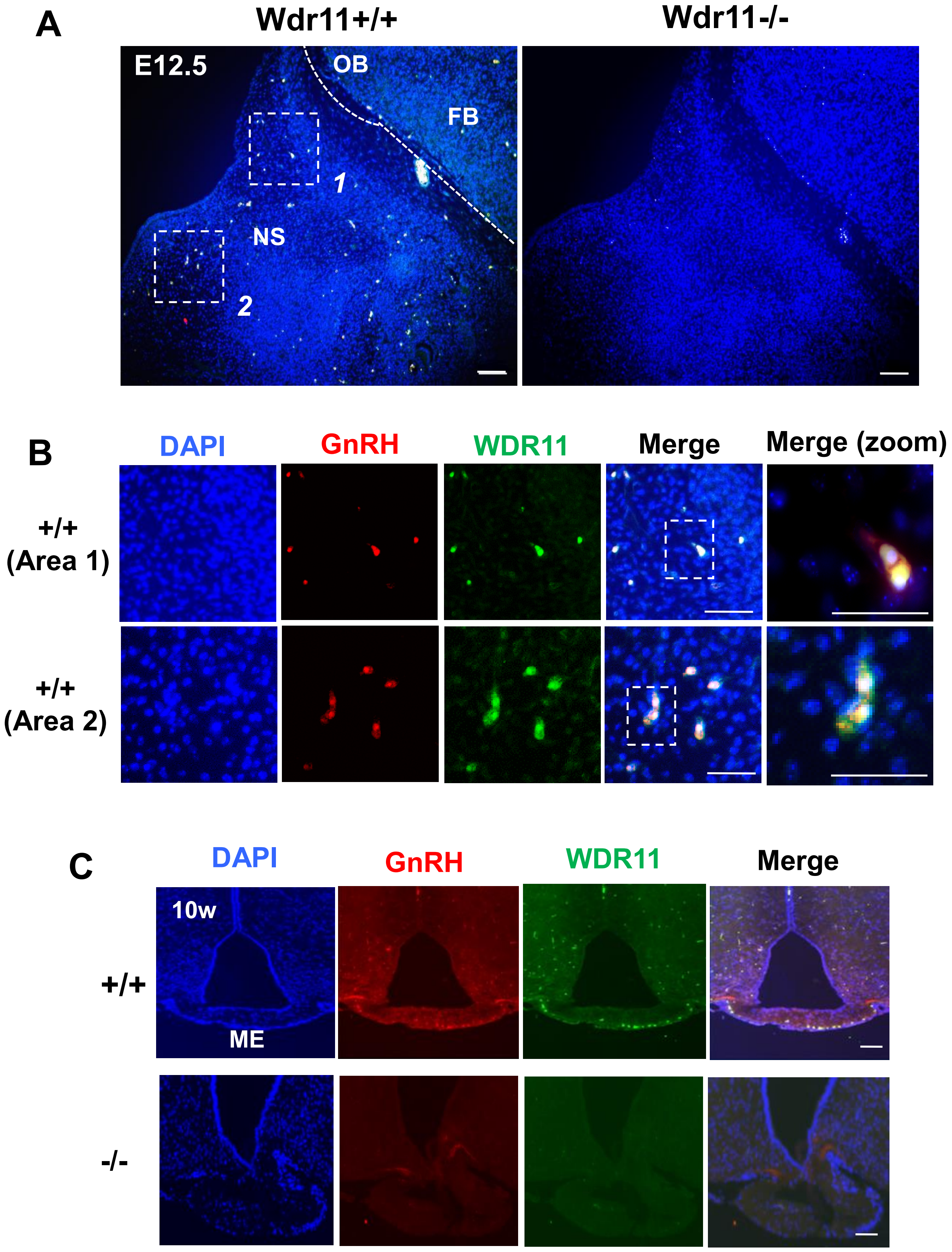

Appendix Figure S3.

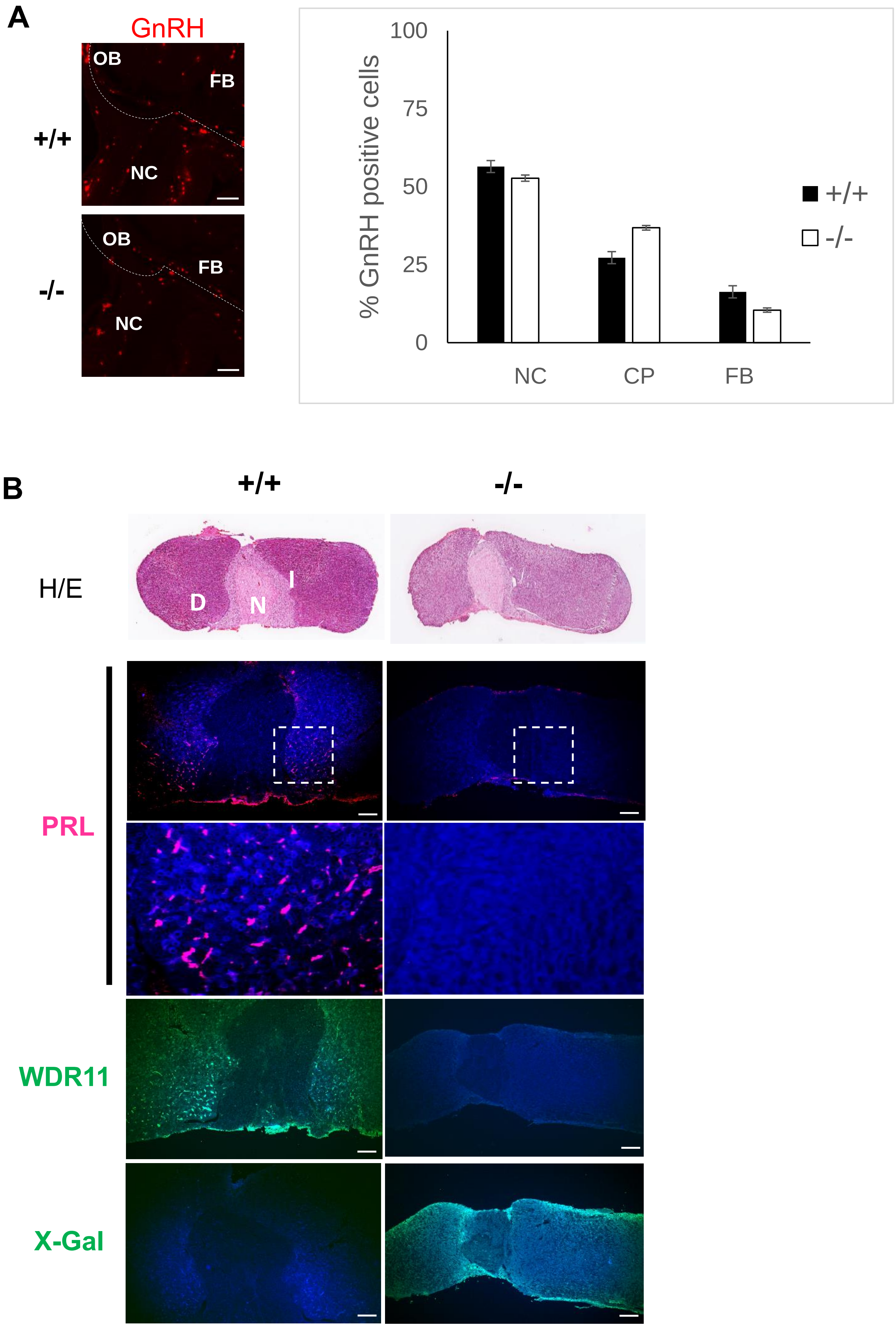

Appendix Figure S4.

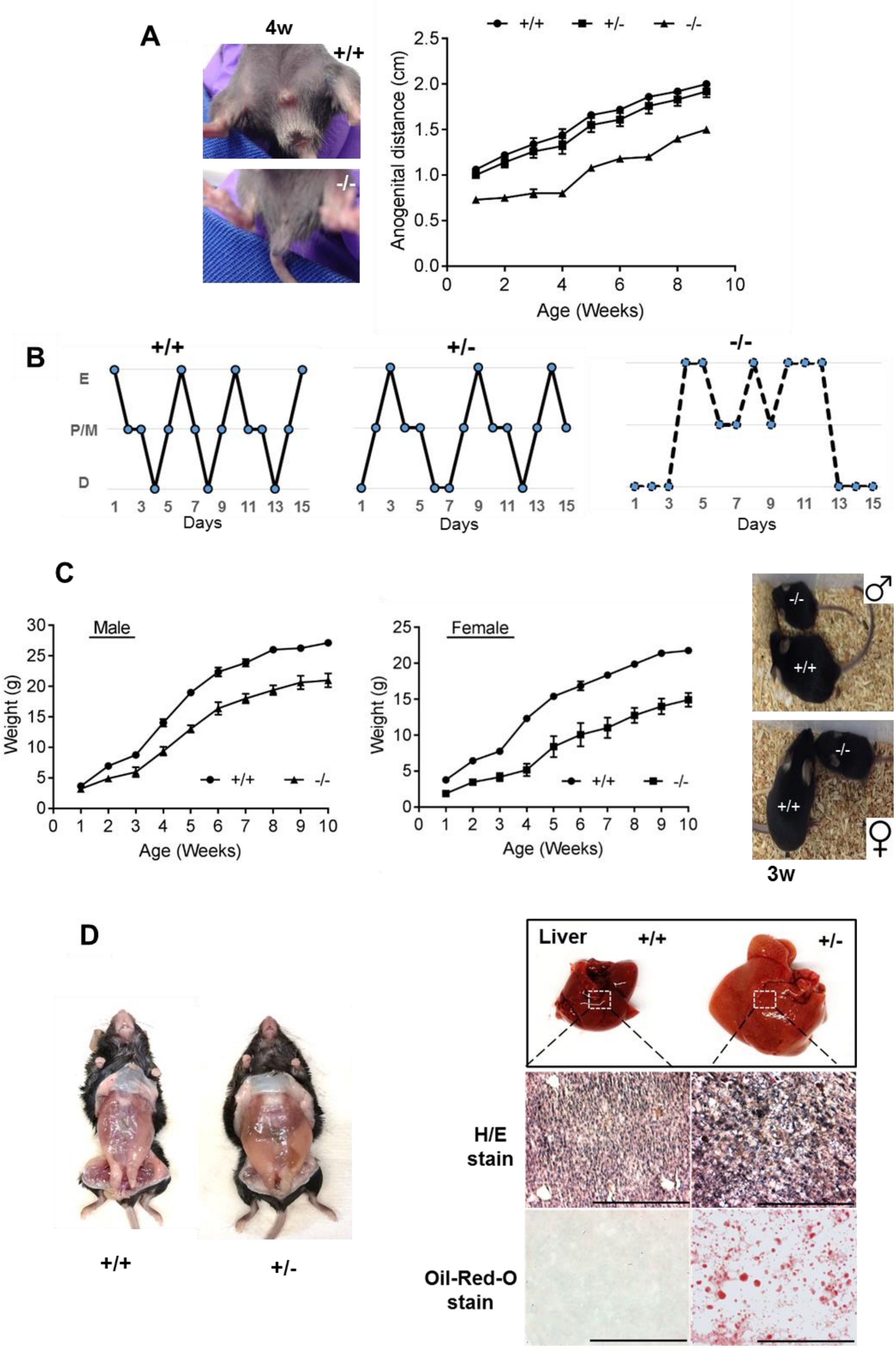

Appendix Figure S5.

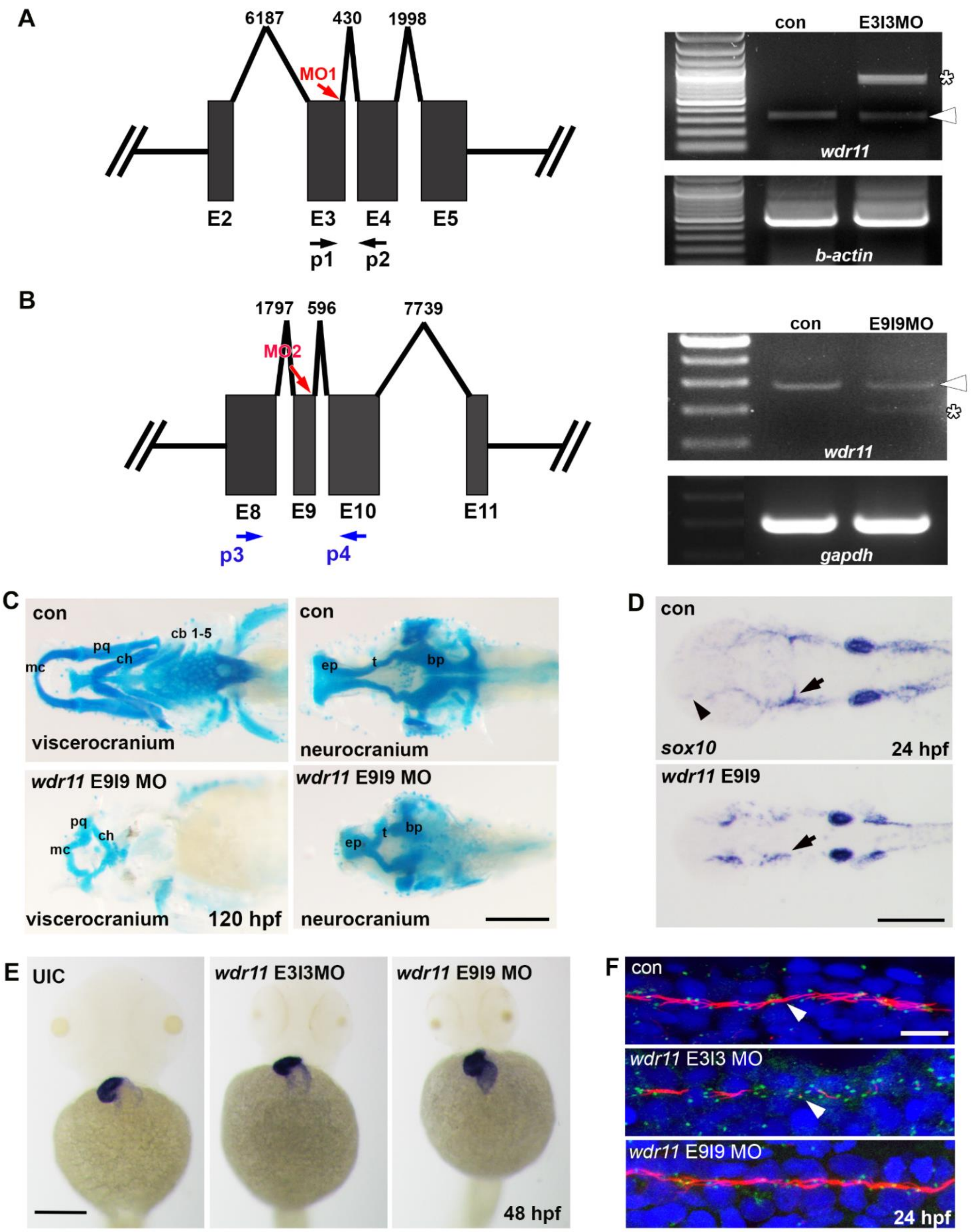

Appendix Figure S6.

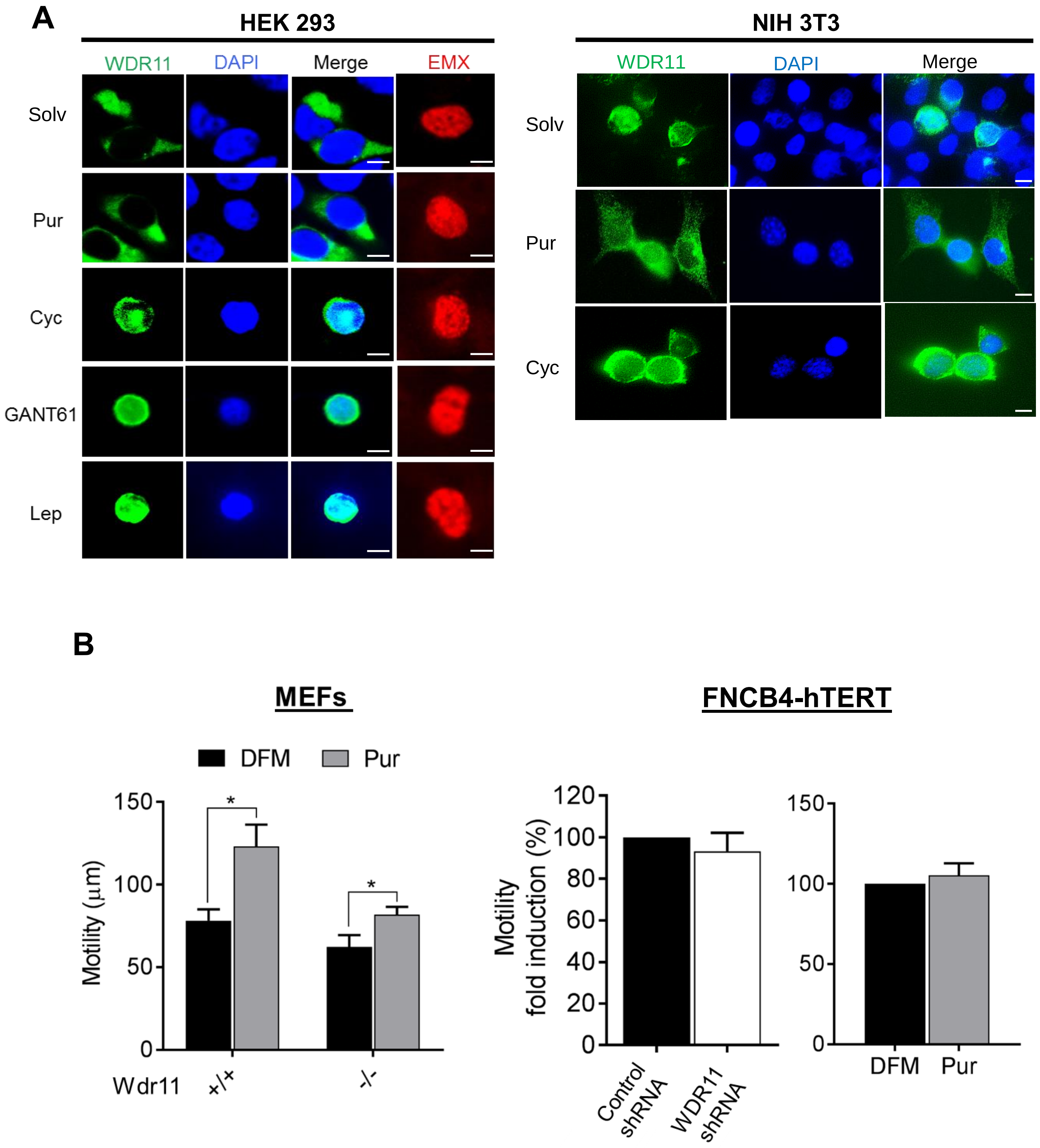

Appendix Figure S7.

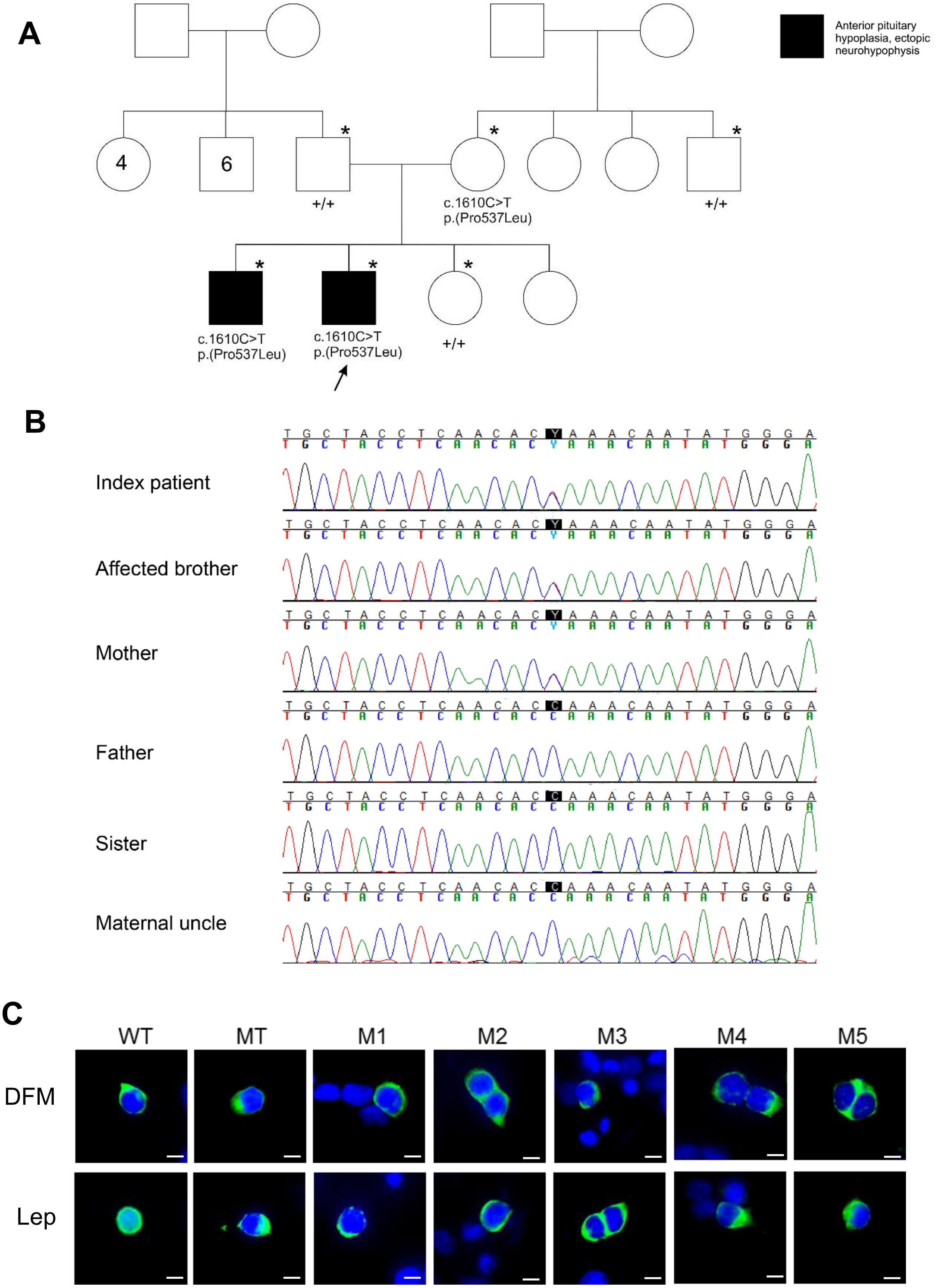

Appendix Figure S8.

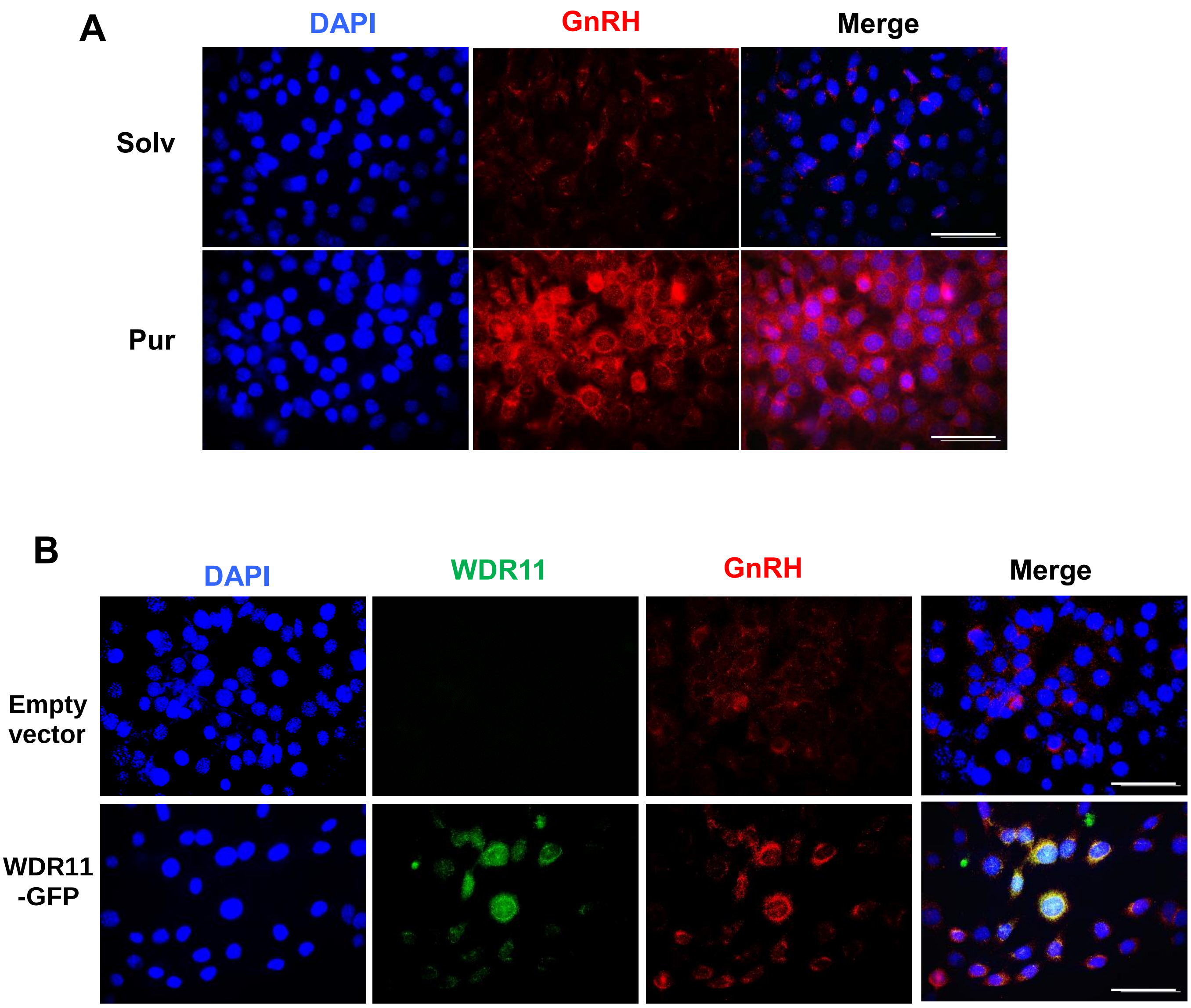

Appendix Figure S9.

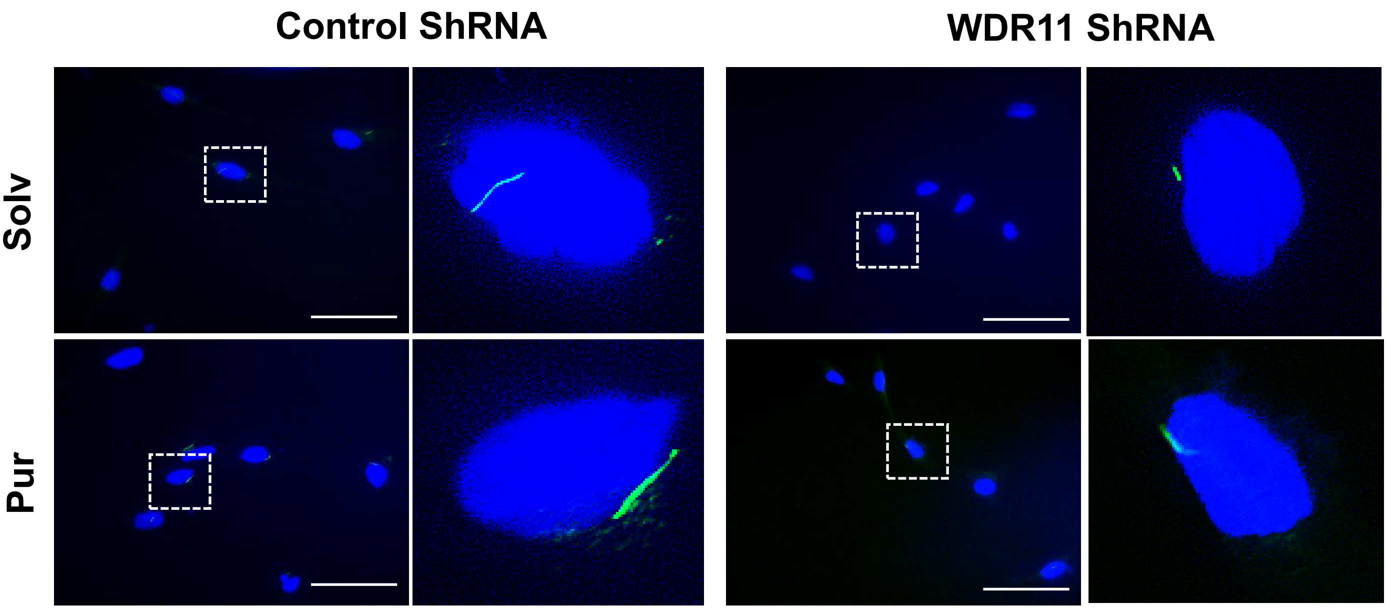

Appendix Figure Legends

Appendix Figure S1. *Wdr11* knockout strategy and expression profiles.

(**A**) The gene trap vector and targeting strategy that disrupted mouse *Wdr11* gene is shown. The PCR primers used to confirm the integration site in exon 3 of *Wdr11* genomic locus are depicted (not in scale). The primer sequences are shown in Supplementary Table 1.

(**B**) RT-PCR analysis of the *Wdr11* heterozygote and null mouse brain tissue confirmed that the expression of *Fgfr2* and *Emx2* was not disrupted.

(**C**) X-gal staining of heterozygote (*Wdr11+/-)* embryos at E10.5 verifying the expression of beta-galactosidase and neomycin phosphotransferase fusion reporter (β-geo-lessCpG) under the endogenous *Wdr11* promoter. The data confirmed a consistent expression pattern of the endogenous *Wdr11* protein and the reporter, which also validated our antibody specificity. Abbreviations are H, heart; BA, branchial arch; MD, mesonephric duct; mes, head mesenchyme; E, eye; FB, forebrain; OB, olfactory bulb; NC, nasal cavity; CP, cribriform plate. Scale bar, 1mm in the whole embryo image, 500μm in the zoomed images.

(**D**) RT-PCR analyses of adult mouse organs using primers for *Wdr11*, *Emx1* and *Emx2* indicate a broad expression profile of *Wdr11*, but more restricted tissue specific expression of *Emx1* and *Emx2*.

(**E**) Various mouse organs dissected from adult and embryos of WT and heterozygotes after x-gal staining.

Appendix Figure S2. *Wdr11* co-localises with GnRH neurones.

(**A**) *Wdr11* is expressed in the GnRH neuronal migratory niche including the nasal cavity and nasal septum, which was absent in the null mice. The zoomed images of dotted areas are shown below. Abbreviations are FB, forebrain; OB, olfactory bulb; NS, nasal septum. Scale bars, 500μm.

(**B**) *Wdr11* co-localises with GnRH positive neurones in the nasal areas of E12.5 embryo brain. Scale bars, 500μm (main image) and 1mm (zoomed image).

(**C**) *Wdr11* is expressed in the median eminence (ME) of 10 week old adult brain. Scale bars, 500μm.

Appendix Figure S3. ***Wdr11*** **KO disrupts the embryonic migration of GnRH neurons and pituitary hormone production.**

(**A**) Quantification of the distribution GnRH neuron in the E12.5 brain. In Wdr11-/-, the relevant proportion of GnRH-positive immunoreactivity was 9.5% higher below the cribriform plate area but 5.9% lower in the forebrain, when compared to WT (n=3). Data are presented as means±SEM. Abbreviations are OB, olfactory bulb; FB, forebrain; NC, nasal cavity.

(**B**) Immunostaining of pituitary gland of 10 week old mice demonstrating the defective expression of prolactin (PRL) compared to the WT. WDR11 and X-gal staining are included as a positive and negative control. Abbreviations are D, pars distalis; I, pars intermedia; N, pars nervosa. Scale bar 100 µm.

Appendix Figure S4. ***Wdr11*** **mutant mice show delayed growth and development, reproductive dysfunction and obesity.**

(**A**) Photographs of external genitalia of male mice. Anogenital distances measured weekly for 10 weeks are plotted as mean±SEM (+/+, n=5; +/-, n=5; -/-, n=5). One-way ANOVA indicates a significant difference (P=0.0034; $F_{(2,24)}$=7.27).

(**B**) Estrous cycling was monitored daily from vaginal smears of 10 week old females. Abbreviations are E, estrous; P, proestrous; M, metestrous; D, diestrous.

(**C**) Body weights of WT (male, n = 5; female, n = 7) and *Wdr11*-/- (male, n = 5; female, n = 5) mice were measured weekly for 10 weeks. Data are presented as mean±SEM. Unpaired student's t-test indicated a significant difference in female (P=0.030114; $F_{(9,9)}$=1.98), but not in male. The photographs of 3 week old mice are shown for comparison of their body length and size.

(**D**) Photographs of 25 week old male mice showing increased accumulation of fatty tissue under the flanks and enlarged liver in the heterozygotes, compared to the WT. Representative images of HE and Oil Red O-stained liver sections revealed the characteristics of fatty liver such as vacuolated hepatocytes containing microvesicular fat. Scale bar 500µm.

Appendix Figure S5. Knockdown of *wdr11* in zebrafish.

(A) Schematic representation and RT-PCR of the target region of E3I3 splice blocking MO. The predicted size of endogenous *wdr11* is 320bp (arrow head) and an intronic inclusion resulting in a predicted premature stop is 760bp (asterisk). *B-actin* as an internal control. (**B**) Schematic representation and RT-PCR of the target region of E9I9 splice blocking MO causing a predicted exon skip and premature stop. The predicted size of endogenous *wdr11* is 400bp (arrow head) and exon9 skip is 299bp (asterisk). *Gapdh* as an internal control. (**C**) Alcian blue staining of *wdr11* E9I9 morphants in the neuro- and viscera-cranium at 120hpf demonstrates a severe loss of cartilage formation. Abbreviations are mc, Meckel's cartilage; pq, palatoquadrate; ep, ethmoid plate; t, trabeculae; bp, basal plate. Con MO, n=47/47; *wdr11* E9I9 MO, n=46/48. Scale bar, 200μm.

(**D**) *Sox10* expression in control and E9I9 morphants at 24hpf shows aberrant neural crest migration into the cranium (arrows) and olfactory bulbs (arrowhead). Con MO, n=20/20; *wdr11* E9I9 MO, n=19/19. Scale bar, 200μm.

(**E**) Loss of wdr11 expression fails to affect normal looping of zebrafish hearts. *In situ* hybridization for *myl7* in uninjected controls (UIC) and E3I3 and E9I9 morphants at 48hpf. UIC, n=50/50; *wdr11* E3I3 MO, n= 49/49; *wdr11* E9I9 MO, n= 50/50. Scale bar, 200μm.

(**F**) Evaluation of acetylated tubulin (red), gamma-tubulin (green), and DAPI (blue) showed reduced ciliogenesis in the pronephric tubules of E3I3 but not E9I9 morphants at 24hpf. Con MO, n=20/20; *wdr11* E3I3 MO, n=12/20; *wdr11* E9I9 MO, n=17/17). Scale bar, 10μm.

Appendix Figure S6. The effects of Hh signalling in the intracellular localisation of WDR11 and GnRH neuronal cell motility.

(**A**) Immunofluorescence of HEK293 and NIH3T3 cells transfected with either WDR11-GFP or HA-tagged EMX1. At 48 hours post-transfection, cells were treated with the indicated compounds for 10 hours, except Leptomycin B (Lep) which was treated for 3 hours. Scale bar 10μm.

(**B**) MEFs and FNCB4-hTERT were serum starved for 24 hours and treated with 10μM Pur or solvent. Twenty randomly selected cells were tracked over 20 hours. Pur significantly induced cell motility of WT MEFs (P=0.04; *t*=3), which was attenuated in *Wdr11* null MEFs. In contrast, Pur treatment did

not induce the motility of FNCB4-hTERT cells (P=0.52; *t*=0.71). No statistical difference was observed in random motility between the control and *WDR11*-shRNA infected FNCB4-hTERT cells (P=0.49; *t*=0.76). Data are means±SEM of 3 independent experiments after unpaired Student's *t* test (*, $P < 0.05$).

Appendix Figure S7. Pedigree and sequence analyses of WDR11 mutation and the defective intracellular localisation of WDR11 mutants.

(**A**) Pedigree of patient MT with a novel *WDR11* mutation (c.1610C>T, p.Pro537Leu). Individuals who went to MRI and were tested for the mutation are indicated with an asterisk. Index patient is indicated with an arrow. The two boys with phenotypes (filled box) inherited the *WDR11* mutation from their mother who has no phenotypes. The numbers of the father's siblings (sisters and brothers) are shown.

(**B**) DNA sequencing chromatograms of the *WDR11* mutation are shown.

(**C**) Immunofluorescence images of HEK293 cells transfected with GFP-tagged constructs of WT and clinically identified mutations of *WDR11*. At 48 hours post-transfection, cells were treated with Leptomycin B and co-stained with DAPI. The percentage of cells showing either nuclear or cytoplasmic localization of WDR11 were quantified. Data from 4 independent experiments, analyzing 100-200 cells in each experiment (Fig. 7D), with the representative images are shown. Scale bar, 10μm.

Appendix Figure S8. Hh agonist induces GnRH protein expression in vitro.

(**A**) Immunofluorescence images of mouse GnRH neuronal cell line GN11 stained with GnRH antibody after 48 hours treatment with purmorphamine or solvent control. Cells with purmorphamine show increased GnRH protein expression.

(**B**) GN11 cells transfected with either empty vector or WDR11 overexpression constructs were stained with GnRH antibody at 48 hours post-transfection. Cells with WDR11 overexpression show higher GnRH protein expression compared to the untransfected neighbouring cells or empty vector transfected. Scale bar 50μm.

Appendix Figure S9. Hh agonist partially rescues ciliogenesis defects in GnRH neuronal cells in vitro.

FNCB4-hTERT cells infected with either control shRNA or WDR11-shRNA were treated with Purmorphamine (Pur) or the solvent (Solv) for 10 hours before staining with anti-ACT antibody (green) followed by DAPI. Scale bar, 50μm.
