# Supplementary material for: WDR11‐mediated Hedgehog signalling defects underlie a new ciliopathy related to Kallmann syndrome
Source: EMBO Rep. 2017 Dec 20;19(2):269–89. doi: 10.15252/embr.201744632 (PMC5797970; doi:10.15252/embr.201744632)
Supplement: Supplementary file 2 — Expanded View Figures PDF [file EMBR-19-269-s002.pdf]

Expanded View Figures

Figure EV1. WDR11 co-localizes with cilia in various ciliated tissues.

A–C Immunofluorescence images stained for acetylated tubulin (ACT) and WDR11 demonstrate their co-localization in the sagittal sections of 10-week-old brain, especially in the olfactory bulb (A), the sperm flagellum (B) and the coronal sections of 12-week-old brain, showing the hypothalamus and median eminence (C). The zoomed images of the dotted area are shown below. *Wdr11*$^{-/-}$ brain exhibits virtually absent ACT staining in all areas. Abbreviations are GLL, glomerular layer; EPL, external plexiform layer; MCL, mitral cell layer; GCL, granule cell layer; ME, median eminence. Scale bars, 500 μm.

▶

 © 2017 The Authors

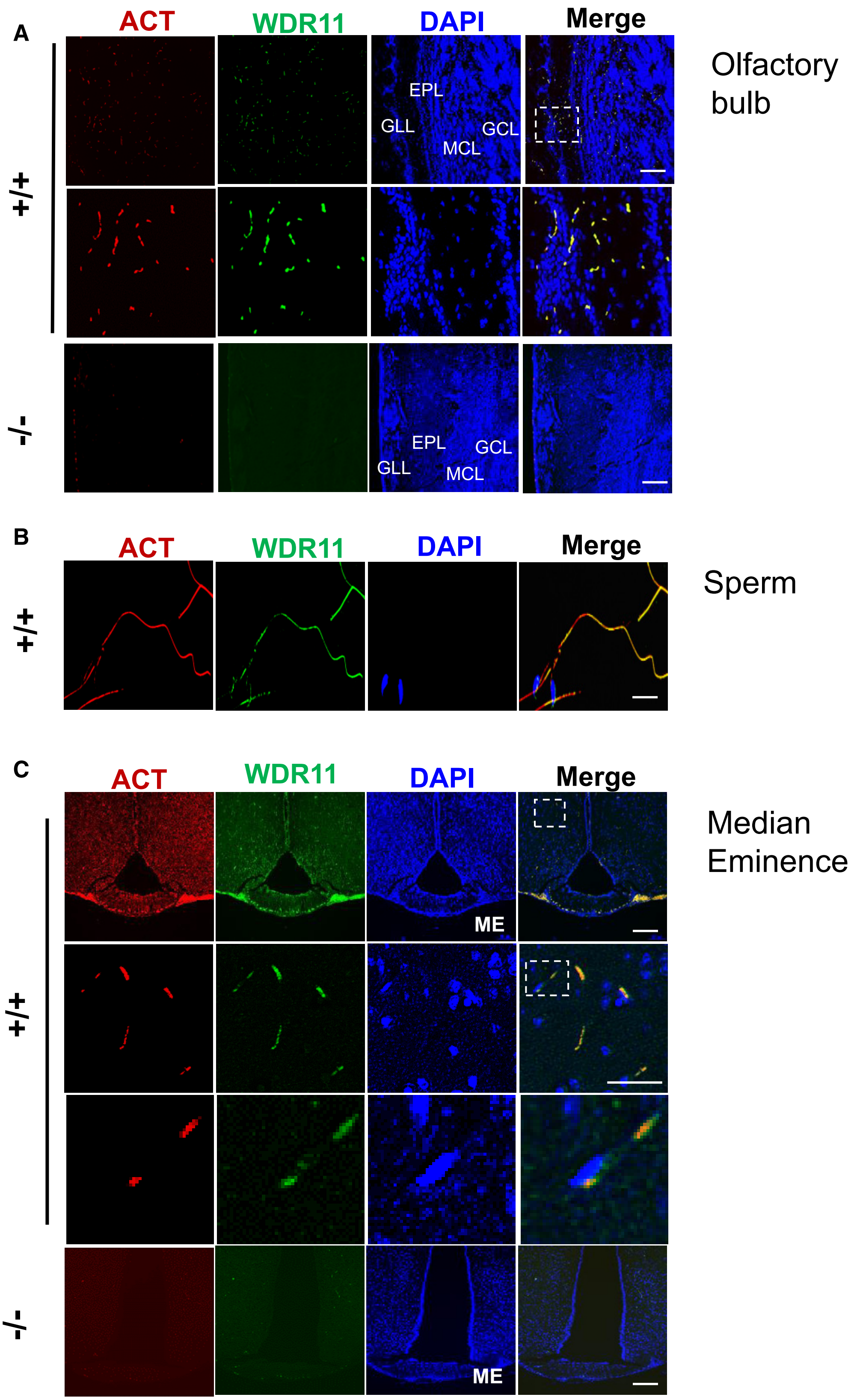
A
ACT
WDR11
DAPI
Merge
Olfactory bulb
+/+
-/-
EPL
GLL
GCL
MCL
B
Sperm
C
Median Eminence
ME

Figure EV1.

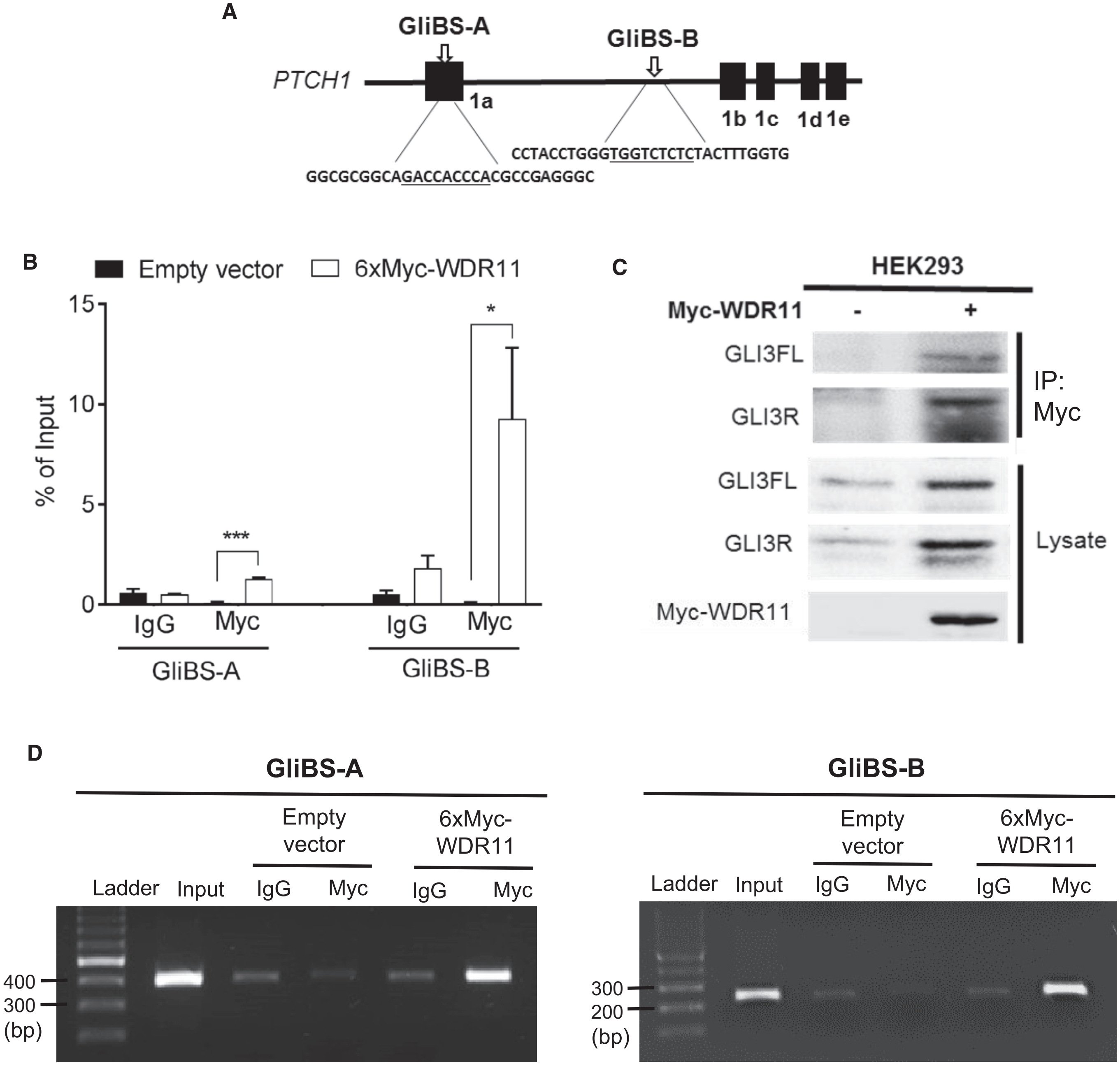

Figure EV2. WDR11 interacts with GLI-binding sites in PTCH1 promoter.

A Schematic diagram showing human *PTCH1* gene locus. The filled boxes indicate the positions of different isoforms of exon 1. Two functional GLI-binding sites (GliBS-A and GliBS-B) are shown with the consensus binding sequences underlined.

B ChIP assay was performed in HEK293 transfected with 6xMyc-tagged WDR11 or empty vector, using anti-Myc antibody or IgG. The qPCR amplification results of the putative GLI-binding sites shown in (A) are shown as % of input (a portion of the sonicated chromatin template before immunoprecipitation). Data are presented as mean±SEM from three independent experiments. Two-way ANOVA followed by Bonferroni's multiple comparison indicated that Myc-WDR11 protein complex was present at both GliBS-A (***P = 0.0002) and GliBS-B (*P = 0.042) sites at significantly higher levels compared to the empty vector-transfected or IgG-immunoprecipitated.

C Co-IP Western blot confirmed the presence of endogenous GLI3FL and GLI3R in the Myc-WDR11 immune complex.

D Agarose gel images showing the PCR amplification products of each target sequence from the input and ChIP samples.

Source data are available online for this figure.

 © 2017 The Authors
