# Supplementary material for: WDR11‐mediated Hedgehog signalling defects underlie a new ciliopathy related to Kallmann syndrome
Source: EMBO Rep. 2017 Dec 20;19(2):269–89. doi: 10.15252/embr.201744632 (PMC5797970; doi:10.15252/embr.201744632)
Supplement: Supplementary file 3 — Movie EV1 [file EMBR-19-269-s003.zip › Movie_EV1_Zip_File/Movie_EV1_Legend.docx]

**Movie EV1 Legend**

**Movie EV1. *Wdr11* KO caused abnormal formation of thoracic skeletons and lung airways.** Movie of the E13.5 *Wdr11-/-* embryo with a transparent thorax and inflated lungs.
