# Supplementary figures and images for: WDR11‐mediated Hedgehog signalling defects underlie a new ciliopathy related to Kallmann syndrome
Source: EMBO Rep. 2017 Dec 20;19(2):269–89. doi: 10.15252/embr.201744632 (PMC5797970; doi:10.15252/embr.201744632)

## Slide 1
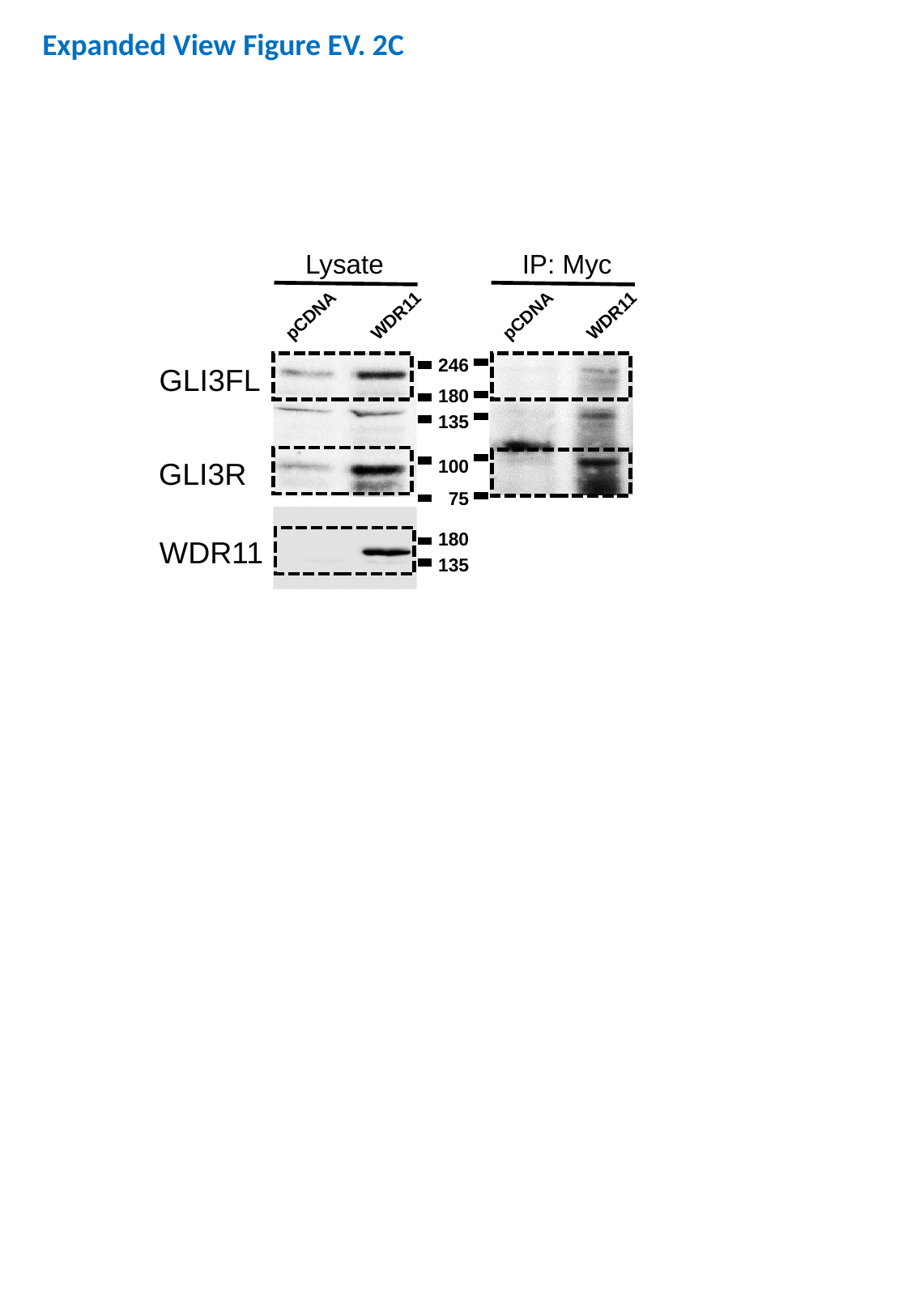

Expanded View Figure EV. 2C
IP: Myc
Lysate
pCDNA
pCDNA
WDR11
WDR11
246
180
135
100
75
GLI3FL
GLI3R
180
135
WDR11

Supplement: Supplementary file 4 — Source Data for Expanded View [file EMBR-19-269-s010.zip › Source_Data_for_Fig_EV2_Kim_et_al.pptx]

## Slide 1
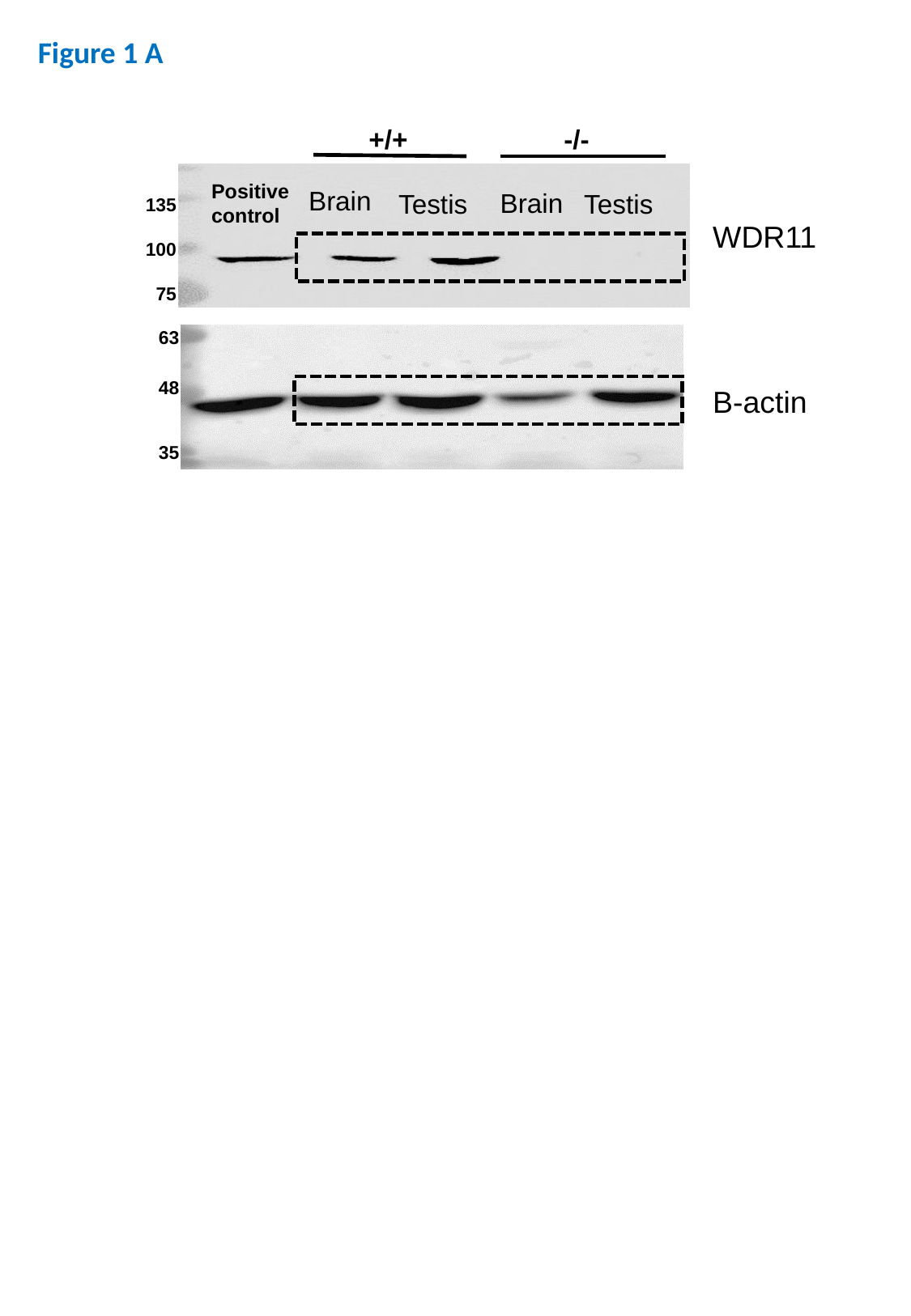

Figure 1 A
-/-
+/+
Positive
control
Brain
Brain
Testis
Testis
135
100
75
WDR11
63
48
35
Β-actin

Supplement: Supplementary file 6 — Source Data for Figure 1 [file EMBR-19-269-s004.pptx]

## Slide 1
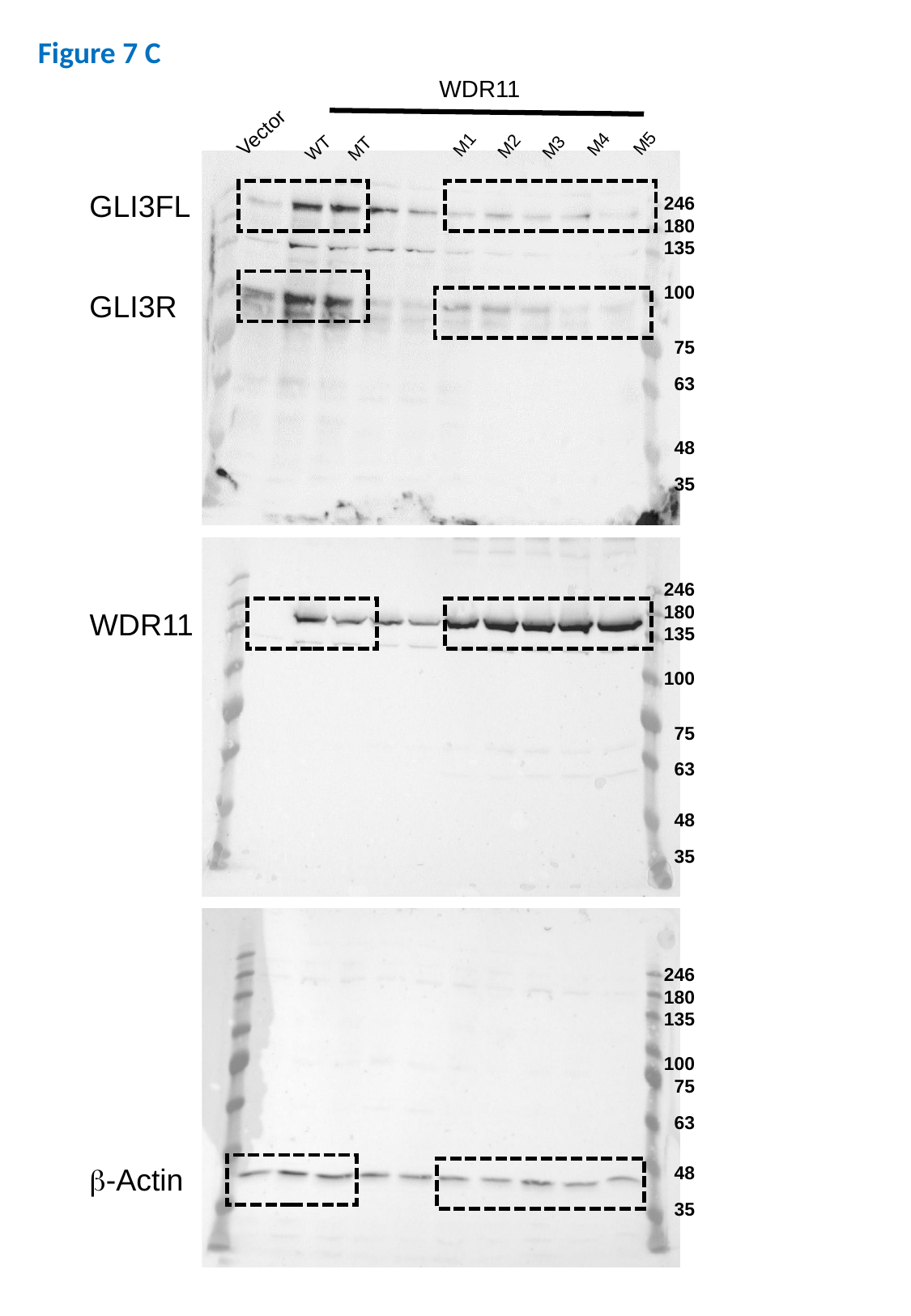

Figure 7 C
WDR11
Vector
M4
M1
M5
M2
M3
MT
WT
GLI3FL
246
180
135
100
75
63
48
35
GLI3R
246
180
135
100
75
63
48
35
WDR11
246
180
135
100
75
63
48
35
b-Actin

Supplement: Supplementary file 10 — Source Data for Figure 7 [file EMBR-19-269-s008.pptx]
