# Supplementary material for: WDR11‐mediated Hedgehog signalling defects underlie a new ciliopathy related to Kallmann syndrome
Source: EMBO Rep. 2017 Dec 20;19(2):269–89. doi: 10.15252/embr.201744632 (PMC5797970; doi:10.15252/embr.201744632)
Supplement: Supplementary file 8 — Source Data for Figure 5 [file EMBR-19-269-s006.pptx]

## Slide 1
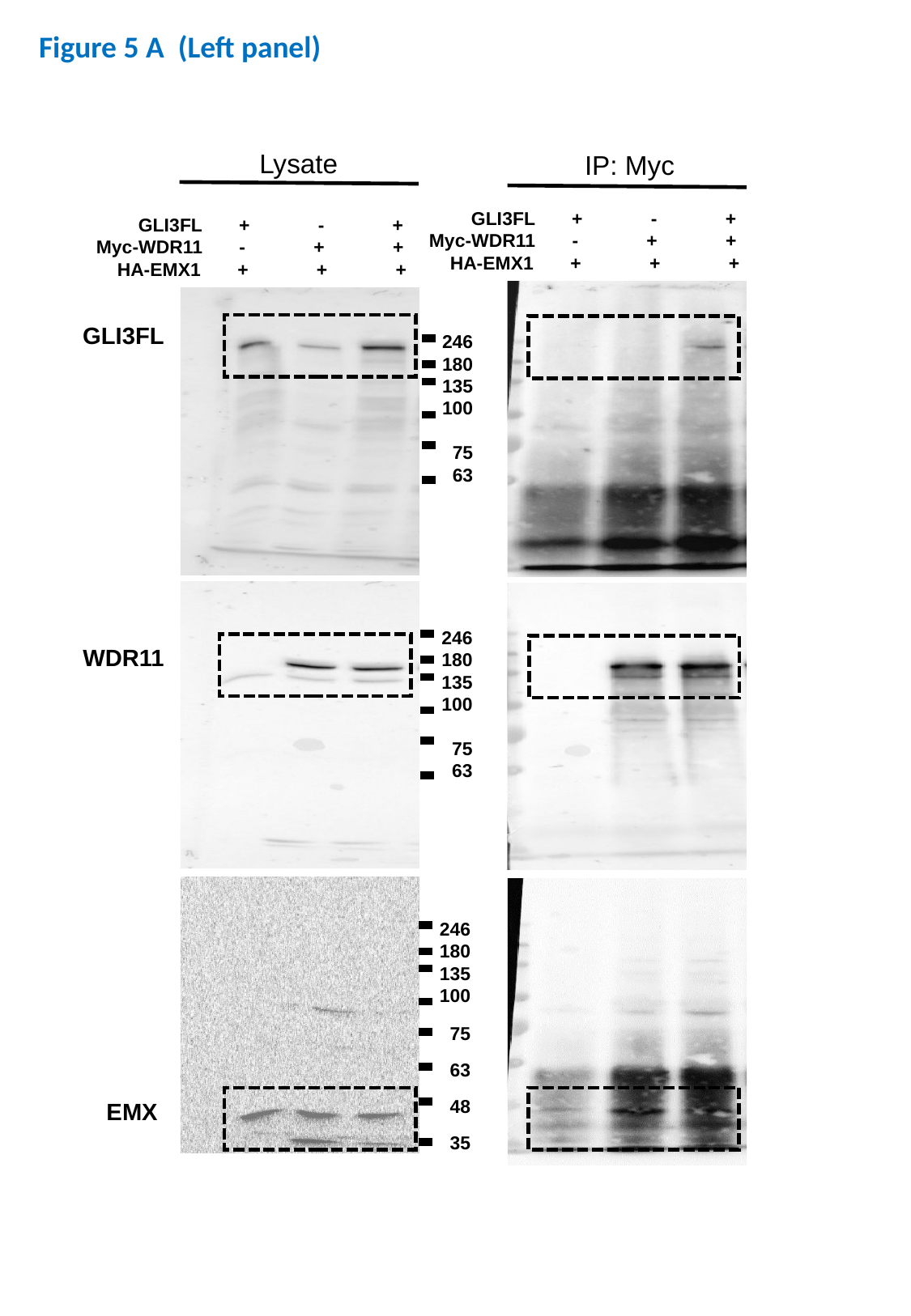

Figure 5 A (Left panel)
Lysate
IP: Myc
 GLI3FL + - +
Myc-WDR11 - + +
 HA-EMX1 + + +
 GLI3FL + - +
Myc-WDR11 - + +
 HA-EMX1 + + +
GLI3FL
246
180
135
100
75
63
246
180
135
100
75
63
WDR11
246
180
135
100
75
63
48
35
EMX

## Slide 2
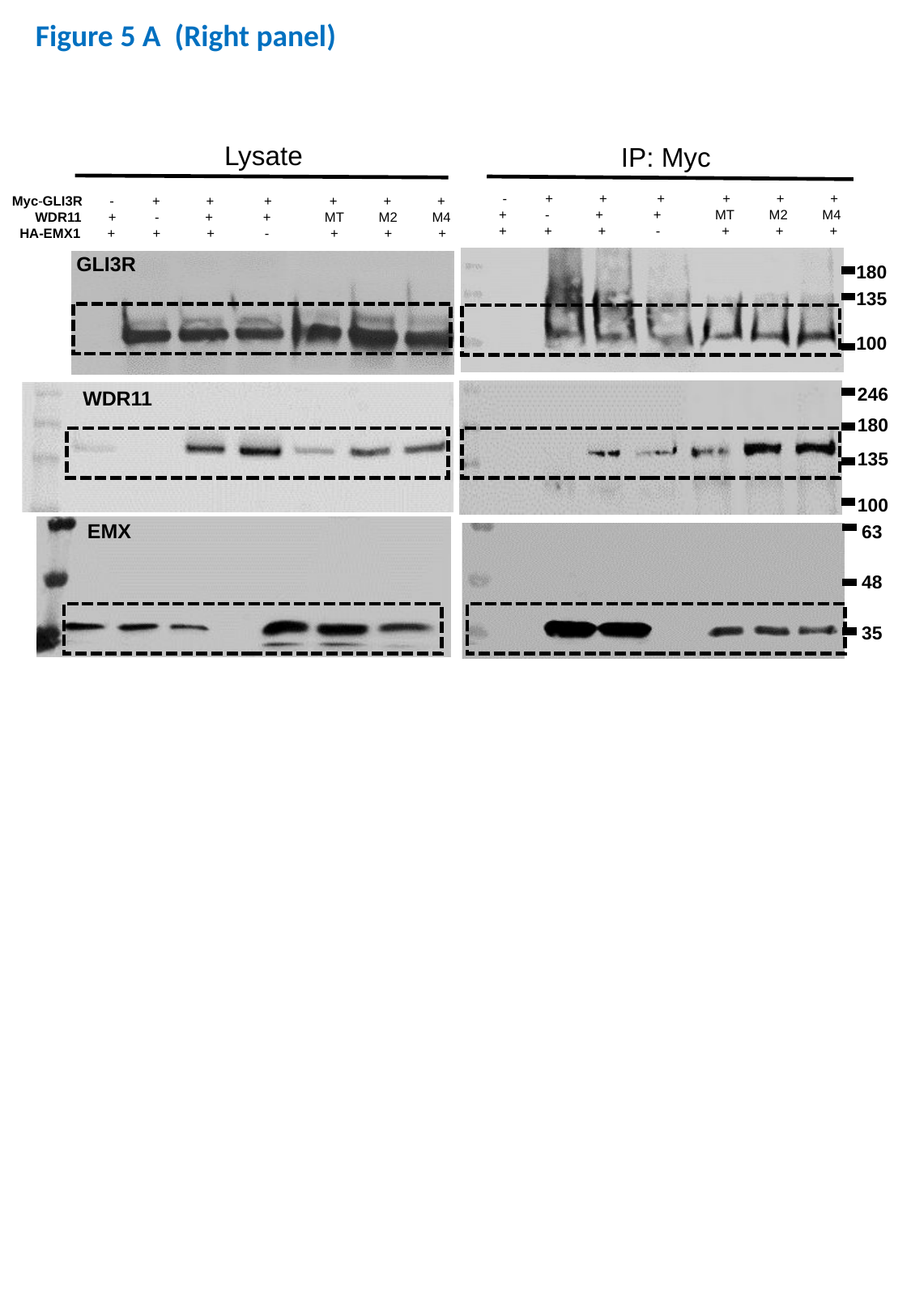

Figure 5 A (Right panel)
Lysate
IP: Myc
 - + + + + + +
+ - + + MT M2 M4
+ + + - + + +
 Myc-GLI3R - + + + + + +
 WDR11 + - + + MT M2 M4
 HA-EMX1 + + + - + + +
GLI3R
180
135
100
246
180
135
100
WDR11
EMX
63
48
35

## Slide 3
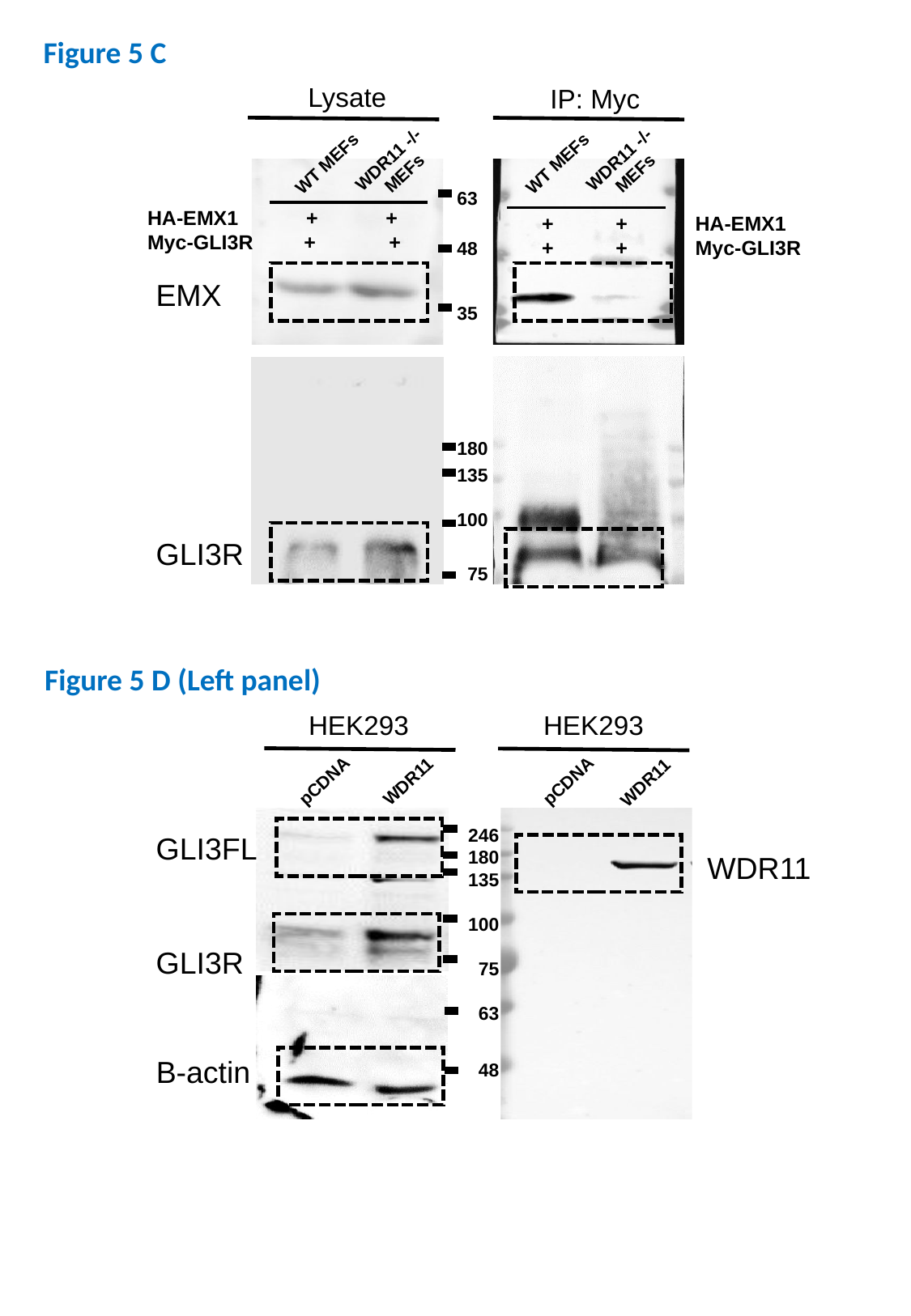

Figure 5 C
Lysate
IP: Myc
WDR11 -/-
 MEFs
WDR11 -/-
 MEFs
WT MEFs
WT MEFs
63
48
35
HA-EMX1 + +
Myc-GLI3R + +
+ + HA-EMX1
+ + Myc-GLI3R
EMX
180
135
100
75
GLI3R
Figure 5 D (Left panel)
HEK293
HEK293
pCDNA
pCDNA
WDR11
WDR11
246
180
135
100
75
63
48
GLI3FL
WDR11
GLI3R
Β-actin

## Slide 4
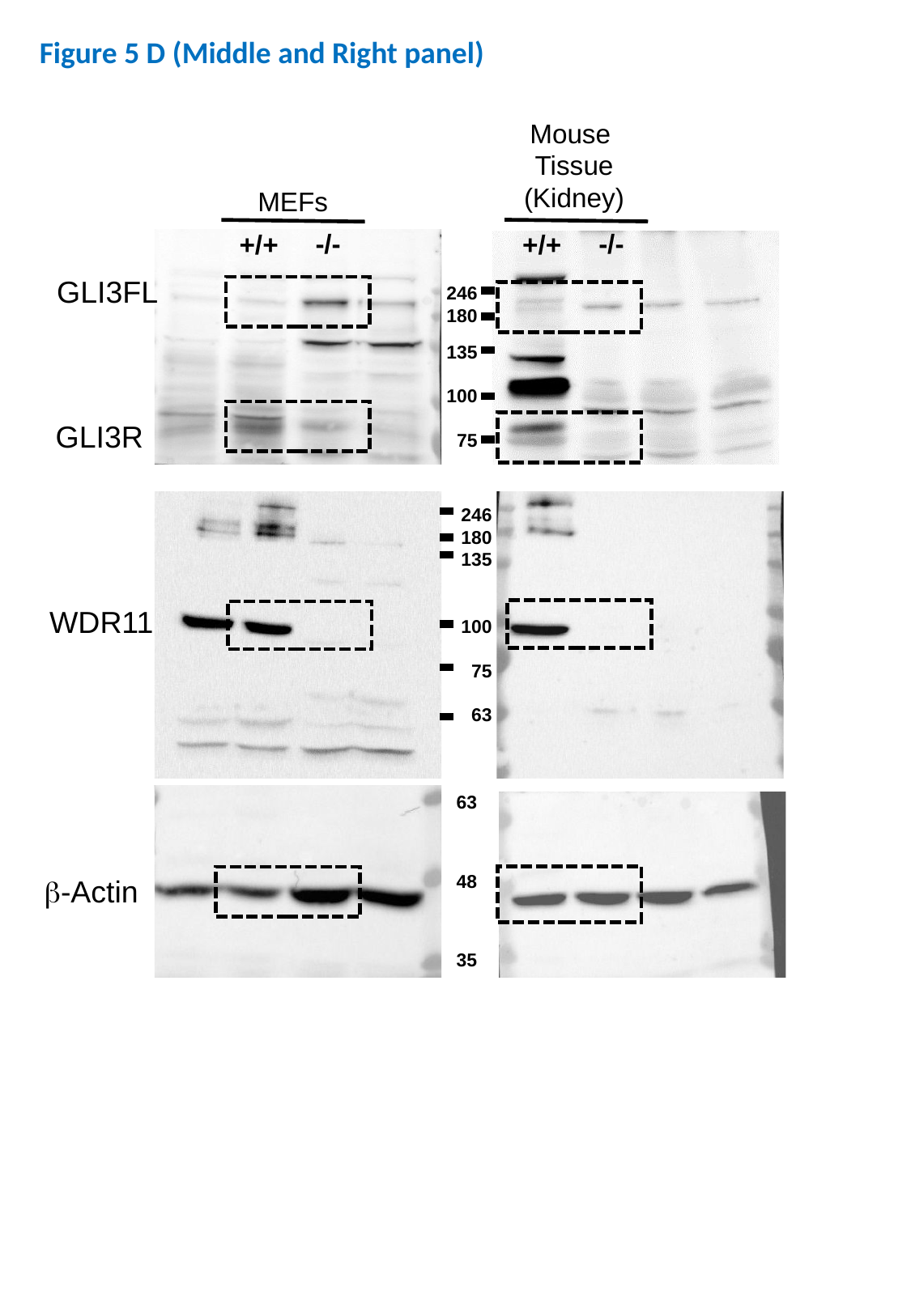

Figure 5 D (Middle and Right panel)
Mouse
Tissue
(Kidney)
MEFs
-/-
-/-
+/+
+/+
GLI3FL
246
180
135
100
75
GLI3R
246
180
135
100
75
63
WDR11
63
48
35
b-Actin
